# Designing Silk-Based Cryogels for Biomedical Applications

**DOI:** 10.3390/biomimetics8010005

**Published:** 2022-12-22

**Authors:** Turdimuhammad Abdullah, Esra Su, Adnan Memić

**Affiliations:** 1 Department of Chemistry, Istanbul Technical University, Istanbul 34467, Turkey; 2 Faculty of Aquatic Sciences, Aquatic Biotechnology, Istanbul University, Istanbul 34134, Turkey; 3 Center of Nanotechnology, King Abdulaziz University, Jeddah 21589, Saudi Arabia

**Keywords:** biomaterials, cryogels, silk, macroporous, composite, tissue engineering, drug delivery, wound healing

## Abstract

There is a need to develop the next generation of medical products that require biomaterials with improved properties. The versatility of various gels has pushed them to the forefront of biomaterials research. Cryogels, a type of gel scaffold made by controlled crosslinking under subzero or freezing temperatures, have great potential to address many current challenges. Unlike their hydrogel counterparts, which are also able to hold large amounts of biologically relevant fluids such as water, cryogels are often characterized by highly dense and crosslinked polymer walls, macroporous structures, and often improved properties. Recently, one biomaterial that has garnered a lot of interest for cryogel fabrication is silk and its derivatives. In this review, we provide a brief overview of silk-based biomaterials and how cryogelation can be used for novel scaffold design. We discuss how various parameters and fabrication strategies can be used to tune the properties of silk-based biomaterials. Finally, we discuss specific biomedical applications of silk-based biomaterials. Ultimately, we aim to demonstrate how the latest advances in silk-based cryogel scaffolds can be used to address challenges in numerous bioengineering disciplines.

## 1. Introduction

There is a technological gap in developing the next generation of biomaterials that are required to meet the current clinical needs [1]. These advanced biomaterials could propel biomedical research by offering unique and improved properties over their current counterparts. Traditionally, hydrogels, or polymeric networks able to hold high amounts of water, have been at the forefront of biomedical research due to their physical, chemical, and other unique properties [1,2]. In the past, hydrogels have shown great potential as scaffolds in several bioengineering applications, demonstrating important features, including biocompatibility, biodegradability, and biomimicry [3]. However, one drawback limiting broader hydrogel applications has been due to lack of a highly interconnected supermacroporous (>10 µm) network structure [2,3]. To address these challenges, researchers have turned to alternative fabrication methods for hydrogel synthesis. One alternative method that could be used to improve hydrogel properties is cryogelation or controlled crosslinking of polymeric networks that occurs at subzero temperatures [4]. Although cryogelation dates back as far as the 1940s, it was not popularized until almost 50 years later [5]. Hydrogels that are made using these cryopolymerization or gelation reactions are suitably named cryogels [4,5]. These cryogels scaffolds often exhibit improved properties over their hydrogel counterparts made from similar biomaterial precursors [6]. 

One biomaterial that has garnered a lot of interest is silk [7]. Although silk has been used in the textile industry for thousands of years as a biomaterial, silk was originally used during suturing in several surgical settings [7]. Silk fibers are composed primarily of silk sericin and silk fibroin, the latter accounting for approximately 75% of its content [8,9]. The unique molecular structure of silk fibroin makes it appealing for several biomedical applications. For example, the hydrophobic silk fibroin molecular subunits can crystallize, forming organized β-sheet structures that lead to water insolubility and high strength [9,10]. On the other hand, the hydrophilic blocks of silk fibroin can absorb high amounts of energy and impart high toughness and elasticity [10]. Previously, silk processing has allowed for the fabrication of foams, films, as well as silk woven and non-woven biomaterials [11]. However, silk-based hydrogels, due to their poor network architecture (i.e., lack of super-macroporosity) and mono-functionality, have found limited utility in more complex biomedical settings [9]. More recently, researchers have turned to alternative fabrication methods and chemistries while focusing primarily on silk fibroin protein (i.e., silk sericin was thought to impart toxicity) to overcome these limitations [12]. 

Traditionally fabrication of macroporous silk fibroin gels was achieved by techniques such as salt leaching, gas foaming, and freeze-drying [12]. However, salt leaching and gas foaming usually require additional processing to remove residual porogens, which could be technically challenging at times [4,12]. On the other hand, recently, silk cryogels have shown great promise in biomedical applications due to their unique properties [8,9,10,11,12]. For example, silk and other scaffolds made by cryogelation exhibit improved physical properties, including shape memory and a highly interconnected macroporous network architecture that supports syringe injectability [11,12,13]. When coupling these improved mechanical properties with the biocompatibility, biodegradability, and biomimicry that silk cryogels offer, such biomaterial scaffolds can truly have multifunctional features [14,15,16,17]. As such silk-based cryogels have propelled many novel bioengineering applications ranging from tissue engineering, wound healing, and drug delivery, just to name a few [9,10,11].

In the past, several notable reviews have been published that cover the use of silk as a biomaterial in general [18,19,20]. However, to the best of our knowledge, there are no review articles that cover the use of silk cryogels specifically for biomedical applications. Therefore, in this short review, we will focus on how silk cryogels are made and how to control their properties for different biomedical applications. Specifically, we will first introduce various parameters that can be used to control the properties of silk cryogels. We will discuss how different crosslinking strategies can be used to tune the physical properties of these gels. Similarly, we will highlight how silk can be combined with other polymers and materials to make composites that further improve scaffold properties and functionality. Next, we will cover specific synthesis parameters that can be advantageous when developing cryogels for specific applications, including cryogels with improved mechanical properties. We will attempt to characterize design principles that lead to functionally improved silk-based cryogels. Subsequently, we will focus on the recent biomedical applications of silk-based cryogels, including their utility in tissue engineering, wound healing, and drug delivery settings. Finally, we will discuss the prospects and future outlook associated with the use of silk-based cryogels, especially in ways that current limitations could be addressed. Ultimately, we hope that this short review will represent an important contribution to the field and propel silk-based cryogel innovation. It is our goal that by highlighting the rational design principles, a wider audience of researchers can investigate novel mechanisms for an array of biomedical applications.

## 2. Fabrication Strategies of Silk-Based Cryogels

Among the two main components of silk, i.e., silk fibroin (SF) and silk sericin, SF has been predominantly used to develop silk-based biomaterials. It should be noted that silk sericin in the past was initially discarded as a biowaste, and recent studies showed its incredible potential in biomedical applications. However, before their use, SF or sericin must be isolated first from the bulk silk to utilize them for developing advanced biomaterials such as cryogels. Despite some modifications in the literature, the method suggested by Kim et al. [21] is generally used to isolate SF from the bulk silk. This method includes removing sericin by boiling the silk in Na_2_CO_3_ solution, solubilizing the silk fibroin in LiBr solution, and dialysis of the fibroin solution against water to remove LiBr [21]. The SF aqueous solution obtained at a concentration of approximately 5% by weight after dialysis can be used in synthesis either directly or by concentrating it further by dialysis against a polyethylene glycol (PEG) solution as needed.

As mentioned earlier, traditional porous material fabrication techniques (porogen leaching and gas foaming) are not preferred due to bio- or cytocompatibility challenges caused by the use of such porogens, as well as the multi-step synthesis conditions [22]. For this reason, cryogelation, which takes place at a lower temperature than the solvent’s freezing point, has been the preferred method to produce three-dimensional (3-D) porous fibroin networks, especially for biological applications [12]. Since this technique enables using ice crystals (i.e., when water is the solvent) as a porogen, it avoids the disadvantages of the traditional methods described above [23]. This method was initially pioneered by Lozinsky et al. to generate macroporous gels with high toughness and super-fast responses [24]. 

**Figure 1:**
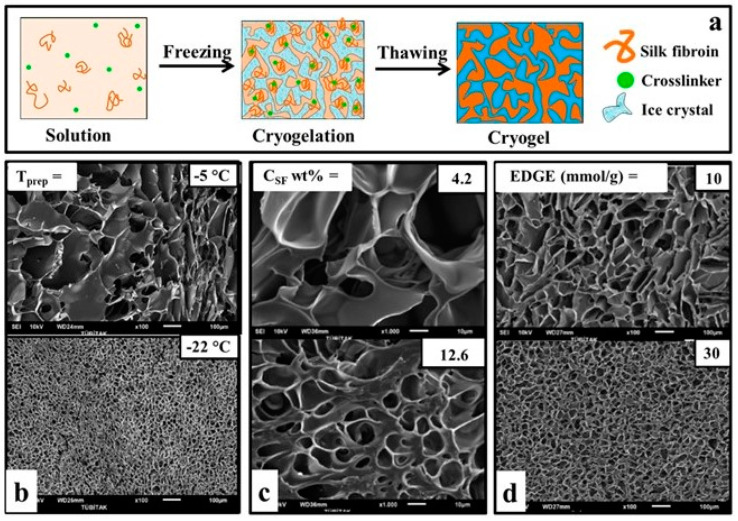
Fabrication strategies of silk-based cryogels. (**a**) SF cryogelation mechanism. (**b**) Effect of temperature on pore architecture of SF cryogels; C_SF_ = 4.2 wt%, EGDE = 20 mmol/g. (**c**) Effect of SF concentration on pore architecture of SF cryogels; EGDE = 20 mmol/g, T= −18 °C. Effect of crosslinker on pore architecture of SF cryogels; C_SF_ = 4.2 wt%, T = −18 °C (**d**). TEMED is constant for all series (**b**–**d**) as 0.25 *v*/*v*%. Scale bars = 100 µm (first and last column) and 10 µm (middle column). (**b–d**) are reproduced from [12] with permission (Copyright © 2013, American Chemical Society).

In this technique, the solutes are stripped from the ice mass and form microchannels by freezing the solution containing the monomer and crosslinker. Thus, crosslinking reactions take place only in these unfrozen regions. The polymeric network, whose microstructure is formed by crosslinking, becomes a macroporous material called cryogels after thawing, which is a negative copy of the ice pattern (Figure 1a) [12]. As demonstrated by Hixon et al. [25], cryogels exhibit much superior mechanical performance over hydrogels. Because cryogelation takes place in concentrated unfrozen microchannels, it allows the formation of macroporous scaffolds with thick pore walls at the end of the reaction [26]. In addition, many cryogels can be compressed even by up to 90% or more without any crack propagation, making them injectable and tough materials [12]. Furthermore, the ability of the cryogels to absorb/release water within seconds and their short response time to stimuli are other critical features that make them superior to traditional hydrogels [26,27,28,29].

### 2.1. Cryogelation Mechanism

Gelation of SF in aqueous solutions occurs through hydrophobic interactions with the formation of β-sheets that will act as a physical crosslink [30,31]. Contrary to the general trend, it is not very accurate to classify SF gels as chemically or physically crosslinked. Because SF molecules form β-sheet nanostructures through physical interactions, but they are essentially linked by irreversible crosslinking sites. This structural transformation (from random coil to β-sheet structure) during gelation can be triggered by various chemical agents (pH regulators, fibroin concentration (C_SF_), cations, diepoxide crosslinkers (1,4 butanediol diglycidyl ether (BDDE), ethylene glycol diglycidyl ether (EGDE)) or physically (i.e. via temperature, vortexing, sonication, and electric field) [21,30,32,33,34].

#### 2.1.1. Chemical Triggers

The most commonly used chemical triggers in SF cryogel synthesis are EGDE, BDDE, and N,N,N′,N′-tetramethylethylenediamine (TEMED). EGDE and BDDE are diepoxy crosslinkers. These attack the amino groups of fibroin molecules to form interchain cross-links and facilitate the conversion from the random coil structure to the β-sheet structure by restricting chain mobility. TEMED is the most commonly used catalyst for SF gelation as a pH regulator. While the gelation of the SF aqueous solution takes days under normal conditions (pH = 5.7), maximum β-sheet (55%) formation is achieved within hours in the pH = 8–9 range with the addition of TEMED. On the other hand, studies of β-sheet formation with the addition of ethanol are frequently performed. The addition of ethanol stimulates the hydrophobic region of fibroin, and water between the chains escapes from the amorphous region. Then, SF random coils form a stable β-sheet structure by rearrangement of hydrogen bonds [35]. Ak et al. [12] fabricated an SF cryogel from 4.2 wt% of SF aqueous solution in the presence of 20 mmol EGDE epoxide/gram fibroin and TEMED at −18 °C. Yetiskin et al. [36] performed a similar cryogel synthesis in the presence of BDDE. However, in their case, the researchers applied directed pre-cooling to the reaction solution and produced anisotropic SF cryogels. On the other hand, Backer et al. [37] stated that they obtained stronger pore walls by treating their collagen, gelatin, and chitosan (CS)-doped SF cryogels with ethanol.

#### 2.1.2. Physical Triggers

When preparing cryogels for biomedical applications having no chemical trigger could be more beneficial, as many of such chemicals could be cytotoxic and additional processes are needed to remove them from the cryogels. Therefore, physical triggers such as sonication, vortexing, and electric field have also been used to fabricate silk-based cryogels [28,34,38]. Among these methods, gelation of SF by sonication is quite interesting, which could produce larger pore sizes and more nested open and closed pores. For example, Hixon et al. [28] applied sonication to 4.5 wt% SF solution for 30 s, and then, the solution was placed at −20 °C for 1 day and thawed at room temperature. Similarly, Kadakia et al. [25] fabricated cryogels from the 4.5 wt% SF solution by sonication. However, they aimed for improved mechanical performance for tissue engineering applications by adding bioactive and osteoconductive precursors (i.e., Manuka honey and bone char) to the structure. 

### 2.2. Parameters Controlling Silk-Based Cryogel Properties

The microstructure of the cryogels, such as pore size, polymer wall thickness, and total porosity, as well as their mechanical properties, can determine their suitability for different applications [22,39]. For instance, large pore sizes provide the cryogels with shorter swelling times and enhanced viscoelasticity that does not allow for physical deformation. In addition, highly interconnected cryogels with suitable pore sizes and wall thickness allow conventional mass transport and facilitate cellular adhesion and migration if they are used as tissue-engineered scaffolds [40]. Therefore, substantial efforts have been made to control pore architecture, mechanical properties, and other important biochemical properties of silk-based cryogels. In the following sections, we compile the literature that studies the effects of cryogelation temperature, cooling rate, solution concentration, and type of crosslinking agents on the properties of silk-based cryogels.

#### 2.2.1. Effect of Cry gelation Temperature

As the cryogelation temperature drops, the high-temperature difference between the solution and the medium leads to faster ice crystal formation, resulting in smaller pore sizes and smoother pores [4,41,42]. Kundu et al. [22] reported that due to their synthesis with 5% by weight SF solution at −20 and −80 °C, the pore size decreased from 75 μm to 58 μm. The authors also reported that total porosity increased with decreasing temperature (from 72% at −20 °C to 84% at −80 °C). In another study, authors reported that the pore diameter of the cryogels obtained at three different temperatures, −5, −10, and −22 °C, decreased from 55 μm to 23 μm (Figure 1b). 

#### 2.2.2. Effect of Cooling Rate

In addition to the effect of cryogelation temperature on the pore sizes, the freezing rate that enables the formation of porogens, i.e., ice crystals, is also a parameter that needs to be examined. Researchers aiming to form anisotropic cryogels with oriented macroporous scaffolds immersed the prepolymer solution in liquid nitrogen (−196 °C) at different rates. By varying the immersion speed in R = 2.5–35 mm/min, the distance between the fibroin layers decreased from 70 µm to 15 µm, while the total porosity remained constant at around 93% [36]. They reported the modulus of the anisotropic SF scaffolds measured in parallel (p) and perpendicular (pp) to the freezing direction. Modulus anisotropy (Ep/Epp), which is used to express the degree of anisotropy, was specified as 4.25 in the study [36]. The same group carried out the same synthesis in a reactor consisting of a copper bottom plate and a cylindrical polytetrafluoroethylene (PTFE) mold, which exhibits a thermal conductivity ratio of 1600, to achieve modulus anisotropy similar to muscles and tendons (Ep/Epp = 20). Moreover, in this way, they obtained scaffolds with the highest modulus anisotropy (Ep/Epp = 21 ± 5) reported to date [43].

In other words, slow cooling rates are required to form large pores, and fast cooling rates are required for small pores. The ice crystals formed during a slow cooling rate grow and lead to interconnected heterogeneous but large pores. At the rapid cooling rate, crystallization will start from more than one point, and small but regular-sized pores will be obtained quickly [36]. Furthermore, since the lower gelation temperature means the reaction solution freezes faster, the reduced pore size with decreasing T_prep_ results in formation of a larger number of tiny ice crystals as the freezing rate increases [4]. As a result, the cryogelation temperature should be chosen as high (i.e. closest to 0 Celsius) as possible to achieve the large pore diameter needed, especially in cell adhesion and proliferation experiments. 

#### 2.2.3. Effect of Polymer Concentration

As the polymer concentration increases, the availability of freezable solvent decreases while the polymer chain entanglement increases during cryogelation, which can lead to the formation of smaller pores and thicker polymer walls [4]. In addition, different conformations of SF could be obtained at the different concentrations, ranging from random coil at the low concentration to type II β-turn and antiparallel β-pleated sheet at the high concentration [30,33]. Furthermore, SF polymer concentration could also influence the solvent freezing rate or temperature through freezing point depression [44]. In the study where the SF concentration was increased from 4.2% to 12.6% by weight, the pore size decreased from 30 µm to 10 µm (Figure 1c). The authors, who changed the C_SF_ between 2.1–16.7 wt% to examine the effect of SF, reported that with the increase in concentration, the pore size, interlayer distance, and total porosity decreased from 93% to 85% [43]. On the other hand, Yetiskin et al. [45] increased the concentration (C_SF_ = 1 to 61.4 wt%) and observed 4 times smaller pore size and porosity due to this lower swelling degree.

#### 2.2.4. Effect of Crosslinking Agents and Condition

To create an interconnected macroporous scaffold during the cryogelation, the polymerization must begin after the formation of ice crystals that act as porogens. If crosslinking occurs faster than the formation of porogens, a nanoporous hydrogel will be produced instead of a cryogel [46]. To prevent this undesirable feature, some inhibitors may need to be added to the system or changes to the polymerization conditions. For example, Ozmen et al. [47] succeeded in delaying the initiation of gelation by adding hydroquinone to the reaction system during the cryogelation of acrylamide-based monomers. The authors stated that by adding 0.2% hydroquinone by weight to the reaction solution, the gelling time of the solution increased from 20 min to 100 min, and at the end of this, they obtained much more homogeneous pores.

Ak et al. [12] carried out the gelation reactions in the presence of EGDE. In this way, the authors stated that they produced macroporous scaffolds with up to 90% porosity and high mechanical strength. They also emphasized that the compressed cryogel swells immediately upon the removal of the load to restore its original shape. In addition, the researchers stated that after using 30 mmol/g EGDE as a crosslinker instead of 10 mmol/g (with respect to silk), the pore size distribution narrowed, causing a more regular pore formation, although there was no significant change in the pore size (Figure 1d). Yetiskin et al. [45] used a successive cryogelation technique to generate SF cryogels with double (DN) and triple networks (TN). They first synthesized the fibroin cryogels (C_SF_ = 4.2 wt%) with single-network (SN) using BDDE as a crosslinker and TEMED as a pH regulator. Then, they performed cryogelation reactions again using SF solution in the presence of BDDE and TEMED in the pores of SN cryogels to create DN and followed the same process to make it TN. The SF cryogels with interconnected DN and TN allowed loading of high SF concentration (above 25 wt.%) and exhibited Young’s modulus of up to 126 MPa and compressive stress of about 240 MPa. They noted that it was 90% compressible, and these values were the highest ever reported for SF-based scaffolds. 

#### 2.2.5. Other Consideration

In addition to the traditional β-sheet formation of SF, chemical and/or surface modifications of SF prior to cryogelation have also been investigated recently. Yetiskin et al. [48] used methacrylated SF (met-SF), instead of pure SF for the cryogel preparation to improve the stretchability and toughness of the cryogels. In their study, glycidyl methacrylate is used for the methacrylation of SF in an aqueous LiBr solution [49]. It has been suggested that the methacrylation of SF facilitates not only the formation of anti-parallel β-sheets together with physical cross-linking but also the formation of covalent bonds within the methacryloyl side [50]. Another method is to rearrange the β-sheet formation of traditional SF by directing chemical side groups and controlling the crosslinking process. For example, Zhang et al. [14] reported that silk cryogels synthesized with amorphous short silk nanofibers (SSF) showed better mechanical properties and cytocompatibility. In addition, the authors emphasized that cryogels obtained with SSF can be functionalized by both hydrophilic and hydrophobic functional groups to make them suitable for a broad range of versatile application.

Overall, low polymer concentration, freezing rate, or high gelation temperature should be preferred in scaffold designs with large macropores and elastic pore walls with injectability and convectional mass transport and cell infiltration applications. The cryogels with higher mechanical strength can be produced by increasing the amounts of crosslinker at low polymer concentration and relatively high gelling temperature to obtain thick pore wall thickness without compromising the pore size. Furthermore, more ingenious cryogelation strategies can be utilized to effectively tune the properties and functionality of silk-based cryogels. 

### 2.3. Silk-Based Composite Cryogels

As stated above, silk-based cryogels have many remarkable features making them attractive for a wide range of biomedical applications. However, as material requirements are increasing, the bar for designing materials for cutting-edge biomedical applications is often high, making it difficult to achieve using a single material source [51,52]. Therefore, natural polymers, including silk, are often combined with synthetic polymers to improve their overall mechanical and structural performance [53,54]. Additionally, SF alone suffers from a lack of inherent antimicrobial and antioxidant properties that could be improved with metal/metal oxide nanoparticles or other antimicrobial/antioxidant agents [55,56]. Therefore, many researchers have turned to having SF or sericin crosslinked incorporating other natural/synthetic polymers, inorganic compounds, and other functional groups to create cryogels with multi-functionality. 

Neo et al. [57] examined the efficacy of composite cryogel with a silk-based composite consisting of polyvinyl alcohol (PVA) and SF. In the study, PVA-SF composite cryogels were produced to eliminate the cell adhesion challenges of PVA cryogels for nucleus pulpous tissue engineering. With the addition of SF, the mechanical strength, as well as the swelling and cell adhesion capacity of the cryogels, were improved. In a study to increase cell adhesion and proliferation in silk fibroin cryogels, biodegradable and biocompatible collagen and its derivative were prepared. Backer et al. [37] found that adding CS to pure silk fibroin not only doubles the porosity of the cryogel but also quadruples the pore surface area. In another study, SF composite cryogel combined with SF, CS, agarose, and hydroxyapatite had enhanced cell adhesion, superior mechanical strength, and bone tissue mineralization properties. Moreover, the authors observed a significant increase in cell viability and proliferation of the composite cryogel when compared to the pure SF cryogel [58]. Yu et al. [59] added tannic acid/ferric ions to create photo-responsive antibacterial CS/SF (CS/SF) cryogels. They compared the SF and CS/SF cryogels and correlated the decrease in pore size with the hydrogen bonds formed in the structure. Another CS/SF cryogel-based study was presented by Han et al. [60] by adding polydopamine (PDA) nanoparticles as a photo-responsive antimicrobial agent to CS/SF cryogels, and they obtained enhanced cell and tissue affinity. On the other hand, Zhao et al. [61] improved the mechanical properties of cryogels used in 3-D tissue regeneration studies by adding cellulose acetate nanofibers (NFs) to CS/SF cryogel scaffolds. Compared to pure scaffolds, the composites have a rough surface, enlarged pore size, and a six-fold increase in compression modulus. Unlike the composite studies described above, Yetiskin et al. [48] have combined the concepts of a DN strategy and composite material by polymerizing acrylic acid and octadecyl acrylate in the pores of SF cryogel. They produced a cryogel with met-SF in the presence of BDDE at −18 °C. The fabricated cryogel was then immersed in a solution of acrylic acid and octadecyl acrylate as monomers, N,N-dimethylacrylamide (DMAAm) as a spacer, N,N’-methylenebisacrylamide (MBAAm) as a chemical crosslinker and ammonium persulfate (APS)-TEMED as a free-radical initiator system. They named this new material class organohydrogel. This new class of materials, containing both hydrophilic and lyophilic phases, is of great interest due to its tunable and programmable mechanical and rheological properties. These materials, which can be easily shaped above the melting point (T_m_ = 49–54 °C) of the crystalline regions they contain, also have shape memory properties.

## 3. Biomedical Application of Silk-Based Cryogels

As a protein-based natural biopolymer approved by the Food and Drug Administration (FDA), silk-based materials have been extensively utilized to fabricate many commercial medical products such as sutures, wound dressing bandages, gels, and masks [55,62,63]. However, emerging biomedical advances, including tissue engineering, controlled drug delivery, and cell-based therapy, require materials with unique structural and physiochemical properties that can hardly be achieved by conventional fabrication techniques [4,64,65]. The cryogelation could surpass these limitations by offering many desirable traits for the silk-based materials, such as a highly interconnected microporous structure, a tunable biodegradation rate, superior mechanical strength, and injectability that greatly extend their application in those advanced biomedical fields (Table 1) [4,12]. In this section, we summarize proof-of-concept studies that have been performed on the clinical usage of silk-based cryogels in tissue engineering, wound healing, drug delivery, and other biomedical application. 

### 3.1. Tissue Engineering

Tissue engineering is currently one of the most rapidly developing technologies that aim to develop engineered tissues consisting of cells, growth factors, and scaffolds to restore the structure and/or function of defected tissues [64]. The scaffold is vitally important in tissue engineering, which should be biocompatible, biodegradable, and its 3-D structure and biomechanical properties should be similar to the native extracellular matrix (ECM) [64,79]. Meanwhile, cell adhesion, growth and migration capacity, oxygen, nutrition transport efficiency, and prevascularization speed are other critical factors in scaffold design [80,81]. Inevitably, silk-based cryogels have been favored for engineering wide ranges of tissue constructs, including intervertebral disc (IVD), cartilage, liver, muscle, and bone [14,22,57]. 

Neo et al. [57] studied the cell attachment capacity, mechanical properties, and surface characteristics of physically cross-linked SF/PVA cryogels towards the regeneration of nucleus pulposus (NP) tissue. They showed that the addition of only 20 wt% of silk significantly improves surface wettability, water adsorption, rehydration capacity, and, eventually, the cell adhesion and proliferation ability of the composite cryogels. Furthermore, the cell-laden SF/PVA cryogels also exhibited compressive modulus and hoop stress values comparable to the native human NP tissue. In another study, Lee et al. [72] investigated SF/PVA cryogels with different mass ratios for cartilage tissue engineering applications through both in vitro and in vivo studies. The cryogels with an equal ratio of SF and PVA displayed the most suitable structural and surface characteristics for the proliferation, migration, and differentiation of chondrocytes to regenerate auricular cartilage. They also fabricated ear-shaped cryogel grafts and transplanted them into rats after seeding rat isolated chondrocytes. The implanted grafts offered enough mechanical strength to retain their shape and phenotypical stability over the culture period and facilitated the formation of a mature cartilage with a lacunar structure without causing any immune response or graft rejection. Li et al. [73] incorporated silver and strontium co-doped hydroxyapatite (AgSrHA) nanoparticles into SF/CS network to formulate a super resilient cryogel that can be used for bone tissue engineering. The presence of silver/ strontium dopants not only improved the cytocompatibility and bioactivity of the cryogel scaffold against bone marrow stromal cells (BMSCs) but also provided a durable antibacterial activity and enhanced osteoinductivity. Furthermore, the bone regeneration efficacy of the developed cryogel was also demonstrated in vivo experiments using a rat skull model. Wang et al. [70] designed a multifunctional scaffold by loading annulus fibrosus cell-derived exosomes into an SF cryogel to seal annulus fibrosus (AF) defects and promote their regeneration. The anti-inflammatory and antioxidant activity, biocompatibility, and fibrocartilage differentiation ability of the designed scaffold were studied by in vitro experiments. The animal experiment further proved that the engineered cryogel scaffold could effectively reverse the degeneration of AF defects in the rat and facilitate their regeneration by regulating the immune microenvironment of the IVD and reducing oxidative stress. Zhang et al. [14] fabricated a series of SSFs injectable cryogels that could be used for engineering wide ranges of tissues. They demonstrated that the mechanical properties of the cryogels can be tuned by adjusting SSFs concentration or crosslinking condition, which can effectively regulate the differentiation behaviors of BMSCs. Moreover, they suggested that the fabricated cryogels can be further functionalized with large variety of bioactive ingredients without compromising their structural and mechanical properties.

**Figure 2:**
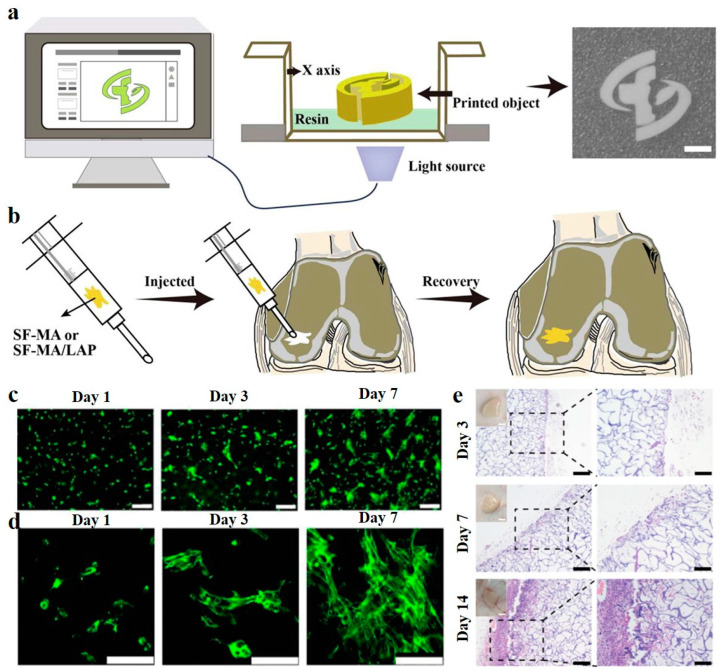
3D Cryoprinting of LAP@SF injectable cryogels for bone tissue engineering. (**a**) Schematic illustration of the printing process of LAP@SF cryogels. (**b**) The printed cryogels can recover their original shape after injection and fit the tissue defect site. (**c**) Fluorescence staining image of live (green)/dead (red) BMSCs, after 1, 3, and 7 days of cultivation on the printed LAP@SF cryogels. Scale bar = 200 µm. (**d**) The morphology of BMSCs, after 1, 3, and 7 days of cultivation on the printed LAP@SF cryogels. Scale bar = 100 µm. (**e**) H&E staining of the printed LAP@SF cryogel scaffold after 3, 7, and 14 days of implantation; left, scale bar = 200 µm; right, scale bar = 100 µm. Reused with some rearrangement with permission of [74] (Under a Creative Commons license: CC BY-NC-ND 4.0).

One of the critical challenges in applying cryogels for tissue engineering is that the shapes of tissue defects are mostly irregular, and by the conventional molding method, it is very difficult to fabricate cryogels that can fill exactly in the defect site [82,83]. To solve this issue, Wang et al. [74] recently presented a 3-D cryoprinting technique to fabricate injectable cryogels composed of laponite nanoparticles (LAP) and SF in predesigned sizes and shapes (Figure 2). In vitro studies and animal model experiments proved that the printed cryogels can promote attachment, propagation, migration, and osteogenic differentiation of the BMSCs. What is more, the printed cryogels can be implanted into the host tissue defects through subcutaneous injection without causing any significant inflammation response, showing their great application potential in bone tissue engineering.

### 3.2. Wound Healing

The effective treatment of non-healing wounds, such as chronic wounds, large or deep wounds, and burn injuries, has been one of the primary concerns in the clinic over the past several decades [84,85]. Such type of wounds require novel bioactive materials that prevent wound infection, ischemia, and oxidative stress and facilitate their re-epidermization by triggering cell proliferation and migration [86,87]. Being a well-known wound healing material since ancient time, silk-based advanced biomaterials in various forms, including cryogels, have shown their incredible potential in the treatment of different types of wounds. For example, Lan et al. [75] studied the burn healing efficacy of an SF-based cryogel embedded with antibiotic gentamycin sulfate (GS)-loaded gelatin microspheres (GM). The SF/GS/GM composite cryogel showed strong antimicrobial activity against several surgical site infection (SSI)-associated pathogens, such as *Pseudomonas aeruginosa* (*P. aeruginosa*), *Staphylococcus aureus* (*S. aureus*) and *Escherichia coli* (*E. coli*). The in vivo rat model study showed that the cryogel dressing could significantly reduce *P. aeruginosa*-associated burn infection and accelerate re-epithelization over the full-thickness burn area. Similarly, Han et al. [60] developed muscle-inspired, antimicrobial, antioxidant, super-elastic, and smart wound dressing by impregnating photo-responsive PDA nanoparticles within an SF/CS cryogel. They proved that the designed cryogel could quickly kill SSI-associated pathogens upon near-inferred (NIR) light exposure and alleviate the oxidate stress at the wound site by suppressing the reactive oxygen species level. In vivo experiment further demonstrated that the combination of cryogel dressing with NIR-assisted photothermal therapy could remarkably accelerate the regeneration of full-thickness epidermal defects. In addition to non-healing wounds, traumatic hemorrhage is another serious global concern, which requires effective hemostatic dressing materials that can rapidly stop bleeding to enhance survival rate [88,89,90]. Recently, Zhu et al. [76] designed an antibacterial and hemostatic cryogel by polymerizing methacryloyl-modified silk sericin, and in situ embedding silver ions into the polymer network at subzero temperature. The cryogel exhibited high blood adsorption capacity, outstanding antimicrobial performance, and much faster hemostatic ability than commercial gelatin in rat tail amputation, liver injury, and femoral artery injury models. They suggested that the rapid hemostatic activity of the cryogel could be due to the high platelets’ adhesion ability of silk sericin.

### 3.3. Drug and Cell Delivery

There have been increasing demands for pharmaceutical products to prevent, treat and cure various diseases by restoring, recovering or modifying organ function [91]. Although pharmaceutical research mostly focused on discovery of new drugs for particular application, much evidence showed that their therapeutic efficiency and side effects strongly depend on the mode of their delivery [92,93]. In this regard, effective drug delivery systems (DDS) often require a biocompatible and biodegradable drug carrier that allows the proper encapsulation of the drugs to prevent them from burst release, control their spatiotemporal delivery, and retain their effective concentration [94,95]. Silk-based cryogels possess the capability to load both hydrophilic and hydrophobic drugs, drug stabilizing ability, and stimuli-responsive properties, making them an excellent DDS [14,62,95]. For example, Zhang et al. [14] showed that hydrophobic curcumin (Cur), which has antioxidant, antibacterial, and anti-inflammatory effects, can be uniformly distributed within the SSF cryogel network. The exposure of Cur-laden SSF to protease XIV caused the degradation of SSF and the sustainable release of Cur to maintain its high antioxidant capacity. In another study, self-assembled SF cryogels were applied for enzyme-responsive delivery of BMSCs-derived exosomes [69]. The integrity and bioactivity of exosomes did not deteriorate during the encapsulation, and their cryogel degradation-dominated release profile was demonstrated both in vitro and in vivo. They also suggested that controlled release of the exosomes could promote cell migration, formation of new blood vessels, and ingrowth of myofibroblasts to improve tissue rehabilitation. 

In addition to being used for DDS, silk-based cryogels have also been utilized to deliver therapeutic cells to diseased organs or tissues for cell-based therapy [77,78]. Tyeb et al. [77] combined stem cell-based therapy with tissue engineering to efficiently repair diabetic wounds. They first fabricated multifunctional scaffolds by coating highly cell-adhesive laminin on antioxidant gelatin-silk sericin cryogels (GSL). Then, adipose-derived stem cells (ADSC) were cultured on the GSL scaffold that can enhance cell proliferation by paracrine factors and differentiate them into fibroblasts, keratinocytes, and vascular endothelial cells to improve the healing efficiency of chronic wounds significantly. In vivo diabetic rat ulcer model study demonstrated that the ADSCs-laden GSL scaffolds exhibit significantly higher cell regeneration, collagen remodeling, and angiogenesis efficacy compared to other control groups.

## 4. Conclusions and Future Direction

In this review, for the first time, we summarize cutting-edge research using silk-based cryogels showing their potential to transform various bioengineering applications. We discuss how purifying silk into its two main constituents can yield biomaterial precursors that can be converted into multifunctional scaffolds. We highlight the advantages of cryogelation as a fabrication method avoiding other more traditional approaches that are plagued with processability challenges that also include generating mechanically inferior scaffolds. Cryogel scaffolds made using various crosslinking mechanisms during cryogelation can be optimized for pore size and network interconnectivity. We show the advantages of chemical versus physical crosslinking and how nanomaterial nanocomposite can be included to further optimize the properties of cryogels and improve their mechanical and other properties. Taken together, this clearly shows the advantage of cryogels over their hydrogel counterparts that exhibit lower mechanical properties, including minimal compressibility, toughness, and the ability to uptake or release water within seconds, quickly respond to external stimuli, and as composites in load-bearing applications. Furthermore, by changing the synthesis parameters, including the temperature of cryogelation and solute concentration, it is possible to truly generate macroporous scaffolds able to undergo syringe injectability, tunable degradation and highly interconnected pore architecture. These properties, therefore, could propel the use of cryogels in a myriad of biomedical applications, including tissue engineering, drug delivery, and wound healing. We finally discussed specific applications where cryogels have unique advantages. For example, silk cryogels have been used in IVD, cartilage, liver, muscle, and bone tissue engineering applications [14,22,57,74]. Cryogels made out of silk-based biomaterials consistently outperformed other scaffolds by offering unique properties where their macroporous structure supports cell growth and migration including nutrient and waste transport, and can improve the speed of scaffolds vascularization [70]. Similarly, in wound healing applications, the combination of silk-based composite cryogels can offer high blood adsorption capacity due to its highly porous features [59,76]. Furthermore, such silk cryogels exhibit outstanding antimicrobial performance with faster hemostatic ability than current commercial products [75,76]. Finally, the unique macroporous cryogel network structure has major advantages for cell and drug delivery [58,69]. For example, silk-based cryogels can be engineered to minimize burst release, provide spatiotemporal control, as well as stimuli-responsive drug delivery of both hydrophilic and hydrophobic drugs [14,62,94,95]. The cryogel macropores likewise allow for the loading of whole therapeutic cells, such as stem cells, into the scaffolds post fabrication that could be used in repairing diseased tissues or organs, including in the treatment of diabetic wounds. 

Although much progress has been made using silk-based cryogels, some challenges still remain. Currently, silk-based cryogels exhibit unique and improved physical properties, but further progress is needed for some complex bioengineering applications. For example, in many biomedical applications, sterilization of the scaffold is required for the clinical application of such scaffolds. While autoclave sterilization has been demonstrated for other cryogel-based scaffolds [29,96], research on silk-based cryogels autoclave processability is lacking. Therefore, currently, some biomedical applications might be out of reach for silk-based cryogels. Similarly, injectable silk-based cryogels need to be improved in terms of their potential stimuli responsiveness using triggers such as oxygen [97], pH [98], and temperature [48] that could propel applications in immunotherapy and cancer treatments. In addition, the use of oxygen-releasing silk cryogels could aid in wound healing applications when combined with improvements in cell adhesion and antimicrobial activity by the use of various silk nanocomposites [16]. On the other hand, when using silk sericin in drug delivery applications, mechanisms that are based on degradation using non-enzymatic approaches of silk could significantly improve their utility [99]. Finally, we were able to review only a handful of references that use silk-based cryogels in 3-D printing applications [74]. Therefore, we believe there is a significant gap in the application of silk-based cryogels when compared to other biomaterial precursors. This includes the application of other natural and synthetic polymers for use in 3-D cryogenic bioprinting for both disease modeling and regenerative medicine [100,101]. To achieve this, making silk-based cryogel bioinks will require more fine-tuning of their mechanical properties that could support high print fidelity and accurate structures reconstruction while retaining their biocompatibility, biodegradability, and biomimicry. In conclusion, silk-based cryogels are unique biomaterials that have the potential to revolutionize many biomedical fields, and we hope for major developments in their application and utility. 

## Figures and Tables

**Table 1:** Biomedical applications of silk-based cryogels.

Composition	Type of Silk	Application	Evaluation Stage	Reference
SF SF	Silk fibroin	Tissue engineering	In vitro	[66]
	Silk fibroin	Liver tissue engineering	In vitro	[22]
SF	Silk fibroin	Tissue engineering	In vitro	[67]
SF	Silk fibroin	Drug delivery	In vitro	[68]
SF	Silk fibroin	Drug delivery/Tissue engineering	In vivo	[69]
SF	Silk fibroin	Regeneration of annulus fibrosus/ Drug delivery	In vivo	[70]
SF	Silk fibroin	in situ tissue engineering	In vivo	[71]
SSF	Silk nanofiber	Tissue engineering/Drug delivery	In vivo	[14]
SF/PVA	Silk fibroin	Regeneration of nucleus pulposus	In vitro	[57]
SF/PVA	Silk fibroin	Cartilage tissue engineering	In vivo	[72]
CANFs@SF/CS	Silk fibroin	Muscle tissue engineering	In vitro	[61]
AgSrHA@SF/CS	Silk fibroin	Bone tissue engineering	In vivo	[73]
SF/CS/AR/BG/HA	Silk fibroin	Bone tissue engineering	In vivo	[58]
laponite@SF	Silk fibroin	Bone tissue engineering	In vivo	[74]
SF/GS/GM	Silk fibroin	Burn healing	In vivo	[75]
PDA@SF/CS	Silk fibroin	Healing of full-thickness wounds	In vivo	[60]
TA/Fe^3+^@SF/CS	Silk fibroin	Traumatic hemorrhage/Wound healing	In vivo	[59]
SMC@Ag	Silk sericin	Traumatic hemorrhage/Wound healing	In vivo	[76]
laminin@Gel/SS	Silk sericin	Cell delivery Healing of diabetic ulcer	In vivo	[77]
laminin@Gel/SS	Silk sericin	Cell delivery/Cardiac repair	In vivo	[78]

SF: Silk fibroin; SSF: short silk nanofiber; PVA: Poly (vinyl) alcohol; CANFs: cellulose acetate electrospun nanofibers; AgSrHA: silver and strontium co-doped hydroxyapatite; AR: Agarose; BG: Bioactive glass; HA: Hydroxyapatite; GS: Gentamycin sulfate; GM: Gelatin microspheres; PDA: polydopamine; CS: Chitosan; TA/Fe^3+^: tannic acid/ferric ion; SMC@Ag: sericin-methacryloyl/silver; Gel: Gelatin; SS: Silk sericin.

## Data Availability

Not applicable.
